# Biogenic silver/silver chloride nanoparticles inhibit human glioblastoma stem cells growth in vitro and Ehrlich ascites carcinoma cell growth in vivo

**DOI:** 10.1111/jcmm.15934

**Published:** 2020-10-13

**Authors:** Syed Rashel Kabir, Zhi Dai, M. Nurujjaman, Xiaoyue Cui, A. K. M. Asaduzzaman, Bin Sun, Xianning Zhang, Hongjuan Dai, Xudong Zhao

**Affiliations:** ^1^ Department of Biochemistry and Molecular Biology University of Rajshahi Rajshahi Bangladesh; ^2^ Key Laboratory of Animal Models and Human Disease Mechanisms of Chinese Academy of Sciences, Kunming Institute of Zoology Kunming Yunnan China

**Keywords:** apoptosis, cell cycle, gene expression, mice, TEM

## Abstract

The importance of biogenic silver/silver chloride nanoparticles has become increasing day by day. In the present study, silver/silver chloride nanoparticles (Ag/AgCl‐NPs) were synthesized from *Kaempferia rotunda* tuberous rhizome extract to evaluate the antiproliferative activity against human glioblastoma stem cells (GSCs) in vitro and Ehrlich ascites carcinoma (EAC) cells in vivo in mice. Synthesis of nanoparticles was confirmed by colour change and UV‐visible spectrum and characterized by TEM, XRD, TGA, AFM and FTIR. *K rotunda* and recently synthesized *Zizyphus mauritiana* fruit extract‐mediated Ag/AgCl‐NPs inhibited 77.2% and 71% of GSCs growth at 32 µg/mL concentration with the IC_50_ values of 6.8 and 10.4 µg/mL, respectively. Cell morphological studies and caspase‐3 immunofluorescence assay revealed that both biogenic nanoparticles induced apoptosis in GSCs. Expression levels of several genes were checked by real‐time PCR after treatment with *K rotunda* tuberous rhizome‐mediated Ag/AgCl‐NPs. PARP, EGFR, NOTCH2 and STAT3 gene expression were decreased with the increase of NFκB, TLR9, IL1, TNFα, IKK and p21 gene that would be the cause of induction of apoptosis in GSCs. The cell cycle arrest at G_2_/M phase was confirmed by flow cytometric assay. Both nanoparticles were injected intraperitoneally to rapidly growing EAC cells for 5 consecutive days. Approximately, 32.3% and 55% EAC cells growth were inhibited by *K rotunda* tuberous rhizome‐mediated Ag/AgCl‐NPs at 6 and 12 mg/kg/day doses, respectively while only 20% cell growth inhibition was monitored at 12 mg/kg/day dose of *Z mauritiana* **‐**mediated Ag/AgCl‐NPs. From the above results, it can be concluded that presently synthesized nanoparticles would be a potent anticancer agent.

## 1 INTRODUCTION

Nanotechnology is widely used in sciences, medicine, biotechnology, electronics and other fields to create numerous novel materials with a variety of applications.^1^ Recently, metal nanoparticles have become increasingly important as alternative antimicrobial and anticancer agents in the biomedical and pharmaceutical sector.^2^ Noble metals such as silver, gold and platinum are used to make the most effective nanoparticles.^3^ Due to the disinfecting nature and tremendous medicinal value, silver has been attracted to the researcher. Silver nanoparticles can be synthesized chemically, electrochemically, photochemically, biologically and by using radiation. Among the techniques, green synthesis of silver/silver chloride NPs (Ag/AgCl‐NPs) gained a lot of interest due to the usage of natural resources, rapidness and eco‐friendliness.^4^ Various biomolecules such as enzymes, proteins, flavonoids, terpenoids and cofactors are present in the fruit, bark, root, and leave of plant those act as reducing and capping agents of silver.^3^, ^4^, ^5^, ^6^, ^7^, ^8^ Some of the synthesized nanoparticles showed potent antiproliferative effect against different cancer cell lines.^9^, ^10^, ^11^, ^12^, ^13^ It was reported that biologically synthesized metal nanoparticles showed genotoxicity that varies case‐by‐case and highly dependent on the synthesis parameters, biological source, applied assay, etc.^14^ Several articles reported that biogenic silver nanoparticles are several times toxic for cancer cells than that of the normal cells^15^ and few of them did not show any toxicity against normal cells.^16^ Although green Ag/AgCl‐NPs were subjected against different cancer cell lines but effects against glioblastoma stem cells (GSCs) in vitro and Ehrlich ascites carcinoma (EAC) cells in vivo were not reported.

Recently, *Zizyphus mauritiana* fruit extract‐mediated Ag/AgCl‐NPs were synthesized at our laboratory and characterized by different spectroscopic methods.^2^ The Ag/AgCl‐NPs exhibited antibacterial, antifungal and cytotoxic effects against EAC and human breast cancer cell (MCF‐7). The cytotoxic effect against MCF‐7 cell was due to the generation of ROS and induction of apoptosis in Fas‐mediated pathway.^2^ Another silver nanoparticle was synthesized by using *Kaempferia rotunda* tuberous rhizome. *K rotunda* is a common medicinal herb in Bangladesh and India. Different phytochemical analysis of *K rotunda* demonstrated the content of many active phytoconstituents, include terpenoid, flavonoid, steroid, alkaloid, crotepoxide, chalcone, quercetin, flavanols, β‐sitosterol, stigmasterol, syringic acid, protocatechuic acid and some hydrocarbons.^17^, ^18^ Two novel lectins with anticancer property were also isolated from *K rotunda* tuberous rhizome.^19^, ^20^

In the present study, we are first reporting biosynthesis of Ag/AgCl‐NPs from *K rotunda* tuberous rhizome extract with the morphological, structural, thermal, functional characterization and surface property. The cytotoxic effects of the *K rotunda* tuberous rhizome and *Z mauritiana* fruit extract‐mediated Ag/AgCl‐NPs were reported against human GSCs in vitro and against EAC cells in vivo in mice.

## 2 MATERIALS AND METHODS

### 2.1 Chemicals and reagents

DMEM, DMEM/F12 medium (1:1), Laminin, Foetal bovine serum purchased from Gibco, MTS from Promega (USA), FITC‐Annexin V/PI from ebioscience (USA), Primary caspase‐3 antibody from Cell Signalling, Cy3 goat antirabbit IgG antibody from Life technology; RT master mix and SYBR green master mix from Applied Biosystem; Primer from TsingKe Biological Technology China; Silver nitrate (Scharlau, Spain). Highest analytical grades of chemicals were used throughout the experiments.

### 2.2 Sample preparation

*K rotunda* tuberous rhizomes were collected from the local market. After washing with deionized water, rhizomes were cut into small pieces and homogenized with deionized water at 1:5 ratios (w/v). Then pH of the homogenized sample was adjusted around 7.0 with 1.0 M Tris‐HCl buffer and centrifuged at 10 000 *g*. The clear supernatant was used for silver nanoparticles synthesis and lyophilized by freeze dryer for cytotoxicity test.

### 2.3 Synthesis of Ag/AgCl‐NPs and analysis of UV‐visible spectra

For the synthesis of silver nanoparticles, 1.0 M of AgNO_3_ was added to the clear supernatant of *K rotunda* tuberous rhizome extract to a final concentration of 1, 2, 3, 4 and 5 mM and kept in sun light for 3 hours. The sample colour changed to brown and deep brown and then analysed by UV‐visible spectrophotometer (Hitachi U‐1800, Japan) at the wavelength range from 200 to 700 nm. At the concentration of 5 mM of AgNO_3_ best result was observed. For the large‐scale synthesis, 250 mL of *K rotunda* transparent supernatant was taken in a 500 mL beaker and 0.5 M of AgNO_3_ was added to reach the final concentration of 5 mM. After that the mixture was kept in sunlight for 3 hours and a deep brown colour was developed. Then the mixture was centrifuged at 10 000 *g* for 30 minutes at 4◦C. Afterwards deionized water was used to rinse the pellet several times. Lyophilized colloidal nanoparticles were used only for the concentration determination and some characterization. The colloidal solution was used for further characterization and biological application. *Z mauritiana* fruit extract‐mediated Ag/AgCl‐NPs were synthesized as described Kabir et al.^2^

### 2.4 Characterization of Ag/AgCl‐NPs

Transmitted electron microscope (TEM, JEOL, Japan) was used to observe the morphology of synthesized Ag/AgCl‐NPs. ‘ImageJ’ software was used to detect the size of synthesized Ag/AgCl‐NPs. X‐ray powder diffraction (XRD) was used to know the structure of the synthesized Ag/AgCl‐NPs by PANalytical Empyrean X‐ray diffractometer with a Cu‐Kα (1.5418A) radiation source and the data were analysed by QualX2.0 software. The scanning was performed in the region of 10° to 80°. Thermal stability of Ag/AgCl‐NPs was performed by using thermal gravimetric analysis (TGA, PerkinElmer STA 8000, USA) at a heating rate of 20°C/min under nitrogen atmosphere from 30 to 835°C. Colloidal Ag/AgCl‐NPs were dried in a glass slide and the surface properties were analysed by an atomic force microscope (AFM, Park system XE 70, Korea) where titanium coated nitrate tips were in tapping mode. For Fourier transform infrared spectroscopy (FTIR, Perkin Elmer, Spectrum 100, USA) band, lyophilized Ag/AgCl‐NPs and crude extract were mixed with potassium bromide (KBr) separately and measured at the frequency range from 4000 to 225 cm^−1^ with a resolution of cm^−1^.

### 2.5 Culture of GSCs and EAC cells

The established human glioblastoma stem cells‐3 (GSCs) was used in this study.^21^, ^22^ Briefly, laminin in PBS (1:100) was added to the cell culture petridish or 96 well flat bottom plate and incubated at 37°C for 2.0 hours. Then cells in DMEM/F12 culture medium were seeded and grown in a CO_2_ incubator at 37°C. EAC cells were propagated biweekly at our laboratory as described by Kabir et al.^23^

### 2.6 Cytotoxicity study by MTS assay

GSCs in DMEM/F12 medium were seeded in a 96 well culture plate (2 × 10^4^ cells/well) which was previously treated with laminin and incubated in a CO_2_ incubator. After 24 hours of initial seeding, cells were treated with 2‐32 µg/mL concentration of *K rotunda* tuberous rhizome and *Z mauritiana* fruit extract‐mediated Ag/AgCl‐NPs for 48 hours. EAC cells were collected from mice as described earlier.^23^ Around 1 × 10^5^ EAC cells in DMEM media were seeded in each well of a 96 well cell culture plate. Then treated with 2.5‐40 µg/mL of *K rotunda* tuberous rhizome‐mediated Ag/AgCl‐NPs and *K rotunda* lyophilized sample (300 & 600 µg/mL) at 37°C in a CO2 incubator for 24 hours. Then MTS (3‐(4,5‐dimethylthiazol‐2‐yl)‐5‐(3‐carboxymethoxyphenyl)‐2‐(4‐sulfophenyl)‐2H‐tetrazolium) was added to each well and incubated in the dark for 2 hours. After colour development, absorbance was measured at 490 nm using a plate reader. Cells without Ag/AgCl‐NPs were used as control.

### 2.7 FITC labelled Annexin V and PI staining

Apoptosis assays of Ag/AgCl‐NPs treated and control cells were performed using an FITC labelled annexin V/PI detection kit (ebioscience, USA). At first GSCs (2 × 10^4^/well) were seeded in a 96 well culture plate and after 24 hours, three wells of cells were treated with 6.8 and 10.4 μg/mL concentration of *K rotunda* tuberous rhizome and *Z mauritiana* fruit extract‐mediated Ag/AgCl‐NPs, respectively for 48 hours. Untreated wells with cells were used as control. Cells were stained with Annexin V and PI according to the manufacturer direction. Finally, early and late apoptosis was observed by using a fluorescence microscope (Olympus IX71).

### 2.8 Caspase immunofluorescence assay

GSCs (2 × 10^4^ cells/well) were seeded in a 96 well culture plate. Three wells were treated for 48 hours with 6.8 and 10.4 μg/mL concentrations of *K rotunda* tuberous rhizome and *Z mauritiana* fruit extract‐mediated Ag/AgCl‐NPs, respectively as described above. Remaining three wells without Ag/AgCl‐NPs were used as control. After that, cells were fixed by 4% paraformaldehyde for 1 hour at room temperature and incubated with 0.1% triton X‐100 in PBS for 5 minutes at room temperature and washed thrice with PBS. Subsequently, 200 µL of 10% goat serum in PBS was added to each well and incubated for 1 hour at room temperature. After removal of the serum, cells were washed by PBS and 100 µL of the caspase‐3 primary antibody (400 times diluted by 0.5% tween‐20 and 1% BSA in PBS) was added and incubated for 1 hour at room temperature. Then cells were washed three times with 0.1% tween‐20 in PBS at room temperature. After 10 minutes tween‐20 was removed and 100 µL of Cy3 goat‐anti Rabbit IgG antibody (500 times diluted in PBS) was added to each well in the dark and incubated for 2 hours at room temperature. Finally, cells were washed thrice by 0.1% tween‐20 in PBS for 5 minutes and stained with DAPI in PBS and observed in a fluorescence microscope.

### 2.9 Genes expression analysis by real‐time PCR

For the isolation of RNA, GSCs (3.2 × 10^5^/well) were seeded in 6‐well culture plate and 24 hours later treated with 6.8 μg/mL of *K rotunda* tuberous rhizome‐mediated Ag/AgCl‐NPs as described above. Cells incubated without Ag/AgCl‐NPs were used as control. For the isolation of RNA, cells were washed by PBS and dissolved in Tri‐reagent (Sigma, USA). Then by using trichloromethane, isopropanol and 70% ethanol in different steps RNA was isolated and collected in DNases and RNases free water. After purification of RNA, 18s gene was used for checking the quality and as references to normalize the qPCR data. cDNA was synthesized from an equal amount of Ag/AgCl‐NPs treated and untreated RNA by reverse transcriptase enzyme as described by the manufacturer (Thermo scientific). Samples were prepared by the addition of cDNA, forward and reverse primers (Supplementary Table‐1), water and 2 × Syber master mix as described by the manufacturer (Applied Biosystems). Bio Rad thermal cycler was used for qPCR and the condition was set to 50 and 95°C for 2 minutes each and for 40 cycles, 95°C for 15 seconds and 60°C for 1 minute. Finally, real‐time PCR data were analysed by double delta CT methods using Excel software where 18s was used as reference gene to normalize the data.

### 2.10 Cell cycle analysis

For cell cycle analysis, GSCs were treated for 48 hours with *K rotunda* tuberous rhizome‐mediated Ag/AgCl‐NPs as described above and sample was prepared according to Kabir et al.^24^ Finally, 10 μL of propidium iodide solution (1 mg/mL) was added to the cell suspension and cell cycle was analysed by flow cytometry (BD FACS Calibur, BD Biosciences).

### 2.11 Animals and ethical clearance

Adult Swiss albino mice were collected from the International Center for Diarrheal Diseases Research, Bangladesh. The Institutional Animal, Medical Ethics, Biosafety and Biosecurity Committee (IAMEBBC, IBSc, University of Rajshahi, Bangladesh) approved (number: 102(6)/320/IAMEBBC/IBSc) this research work.

### 2.12 Determination of EAC cells growth inhibition in vivo in mice

EAC cells growth inhibition was examined according to Kabir et al.^24^ In shortly, EAC cells were collected from EAC bearing mice and 1 × 10^6^ cells in 0.1 mL of saline were injected intraperitoneally to 30 Swiss albino mice and kept at room temperature for tumour inoculation for 24 hours. After randomly distributed the mice into five groups (six mice per group), four groups were treated intraperitonially with *K rotunda* tuberous rhizome and *Z mauritiana* fruit extract‐mediated Ag/AgCl‐NPs separately at the doses of 6.0 and 12.0 mg/kg/day for five consecutive days. The remaining fifth group was used as control. On the seventh day of the EAC inoculation each of the mice was sacrificed to harvest a total number of EAC cells in normal saline. Then the cells were counted by a light microscope. Finally, the following formula was used to calculate the percent of growth inhibition.

$$\text{Per cent of inhibition = 100 -}\;\left\{(\text{cells from Ag/AgCl - NPs treated EAC bearing mice/cells from EAC bearing control mice})\times 100\right\}$$

## 3 RESULTS

### 3.1 Synthesis and characterization of Ag/AgCl‐NPs from *K rotunda* tuberous rhizome

**FIGURE 1:**
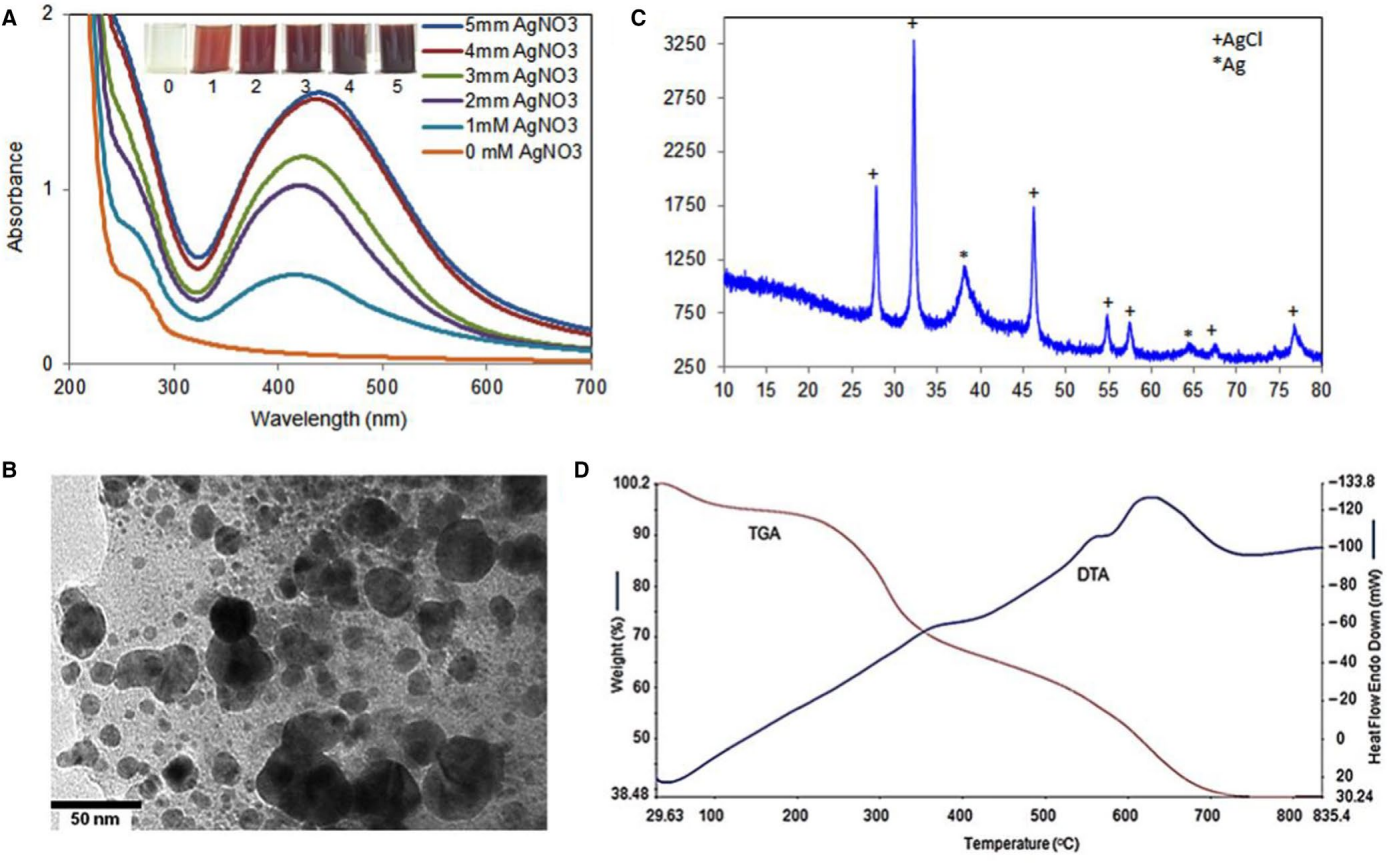
Synthesis and characterization of Ag/AgCl‐NPs. A, UV‐visible spectra of the reaction mixture at different concentrations of AgNO_3_ during synthesis of Ag/AgCl‐NPs; inside of (A) AgNO_3_‐treated and ‐untreated *K rotunda* extracts. Lane 1 *K rotunda* extract without AgNO_3_. Lane 2‐6 indicating *K rotunda* extract treated with 1, 2, 3, 4 and 5 mM of AgNO_3_, respectively. B, TEM micrograph of the synthesized Ag/AgCl‐NPs. C, X‐Ray Diffraction (XRD) pattern of *K rotunda*‐mediated silver nanoparticles, D, TGA micrograph showing the weight loss of Ag/AgCl‐NPs with temperature

Intensity of the deep brown colour formation was checked after incubating of *K rotunda* tuberous rhizome extract with AgNO_3_ at different concentrations. The deepness of brown colour was raised with the increase of AgNO_3_ concentration that indicated the formation of Ag/AgCl‐NPs as shown in Figure 1A. The maximum absorbance peak was observed at 437 nm when the extract was incubated with 5 mM of AgNO_3_ for 3 hours as shown in Figure 1A. About 1.5 g of silver nanoparticles was synthesized from 1.0 kg of *K rotunda* tuberous rhizome with the final concentration of 10 mg/mL.

#### 3.1.1 Morphological characterization Ag/AgCl‐NPs

Highly monodispersed spherical particles were found with a diameter of 3 to 35.0 nm. Maximum nanoparticles were below 10 nm and the average size was obtained 17 nm (Figure 1B).

#### 3.1.2 Structural characterization of Ag/AgCl‐NPs

The XRD pattern of the synthesized Ag/AgCl‐NPs was recorded and the reflection peaks observed (2*θ*) at 27.7°, 32.12°, 46.06°, 54.74°, 57.36°, 67.42° and 76.79° corresponding to the crystallographic planes (111), (200), (220), (311), (222), (400) and (420) respectively, these recognized as AgCl‐NPs (card no. 00‐901‐1666) formation. While reflection peaks 38.04° and 64.36° corresponding to planes (111) and (220), respectively for AgNPs (card no. 00‐150‐9146) as shown in Figure 1C. In both cases crystal systems were cubic.

#### 3.1.3 Thermal characterization of Ag/AgCl‐NPs

The synthesized Ag/AgCl‐NPs lost the weight in three steps that was represented in the TGA plot/profile (Figure 1D). First, second and third weight lost were performed in the temperature range from 30 to 100°C, 100.1 to 350°C and 350.1 to 676°C corresponding to 3.9%, 24.74% and 29.98% weight loss, respectively.

#### 3.1.4 Surface properties of the Ag/AgCl‐NPs

**FIGURE 2:**
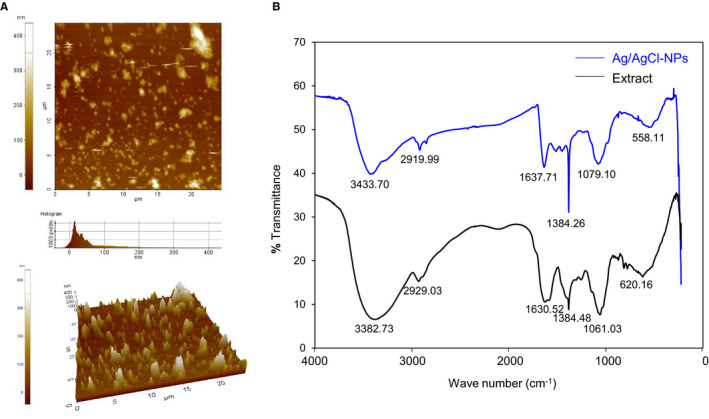
Surface and functional properties of the synthesized Ag/AgCl‐NPs. A, AFM topography of *K rotunda‐*mediated Ag/AgCl‐NPs. Upper and lower represented 2‐dimension and 3‐dimensional views. B, FTIR spectrum of the *K rotunda* extract and synthesized Ag/AgCl‐NPs

AFM was used to determine the dimensions of the synthesized nanoparticles. In the AFM image, the bright spots indicate the presence of Ag/AgCl‐NPs. Whereas, size and dispersion of the nanoparticles represented by the dark spots. The sharp peaks in the 3D image confirm the lower to higher sizes of nanoparticles (Figure 2A).

#### 3.1.5 Functional characterization of Ag/AgCl‐NPs

FTIR spectra of *K rotunda* tuberous rhizome crude extract and Ag/AgCl‐NPs were presented in Figure 2B. The major peaks obtained for crude extract were 3382.73, 2929.03, 1630.52, 1384.48, 1061.03 and 620.16 cm^−1^ whereas for the synthesized Ag/AgCl‐NPs those were 3433.70, 2919.99, 1637.71, 1384.26, 1079.10 and 558.11 cm^−1^.

### 3.2 Effects of Ag/AgCl‐NPs on the cell growth of GSCs and EAC cells

**FIGURE 3:**
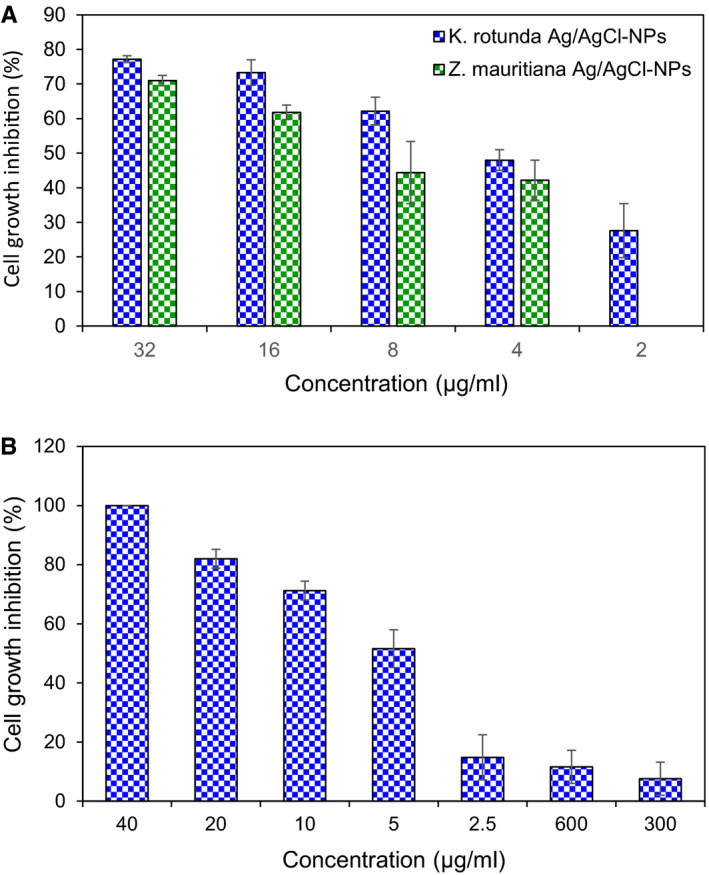
Antiproliferative activities of Ag/AgCl‐NPs. A, GSCs cells growth inhibition by *K rotunda* and *Z mauritiana*‐mediated Ag/AgCl‐NPs in vitro. The cells were incubated with various concentrations of Ag/AgCl‐NPs at 37°C with 5% CO_2_ for 48 h. Then the inhibition ratios were measured by MTS assay (n = 3, mean ± SD). B, EAC cells growth inhibition by *K rotunda* extract (300 and 600) and *K rotunda*‐mediated Ag/AgCl‐NPs

*K rotunda* tuberous rhizome and *Z mauritiana* fruit extract‐mediated Ag/AgCl‐NPs inhibited 77.2% and 71% of GSC cell growth at 32 µg/mL concentration, respectively. The growth inhibition was decreased with the diminish of the nanoparticle concentration as shown in Figure 3A. IC_50_ values of the nanoparticles for GSCs were calculated to be 6.8 and 10.4 µg/mL, respectively. EAC cells growth inhibition was studied after treatment with *K rotunda* tuberous rhizome extract and *K rotunda* tuberous rhizome‐mediated Ag/AgCl‐NPs. Approximately, 14.8% of growth inhibition was observed at the 2.5 µg/mL of nanoparticles. The inhibition increased with the rise of concentration and finally 100% inhibition was observed at 40 µg/mL and the IC_50_ value was calculated to be 7.85 µg/mL. On the other hand, 11.6% and 7.6% of growth inhibition were obtained at 600 and 300 µg/mL of *K rotunda* tuberous rhizome extract (Figure 3B).

### 3.3 Study of Ag/AgCl‐NPs induced apoptosis by Annexin V/PI and immunofluorescence assay

**FIGURE 4:**
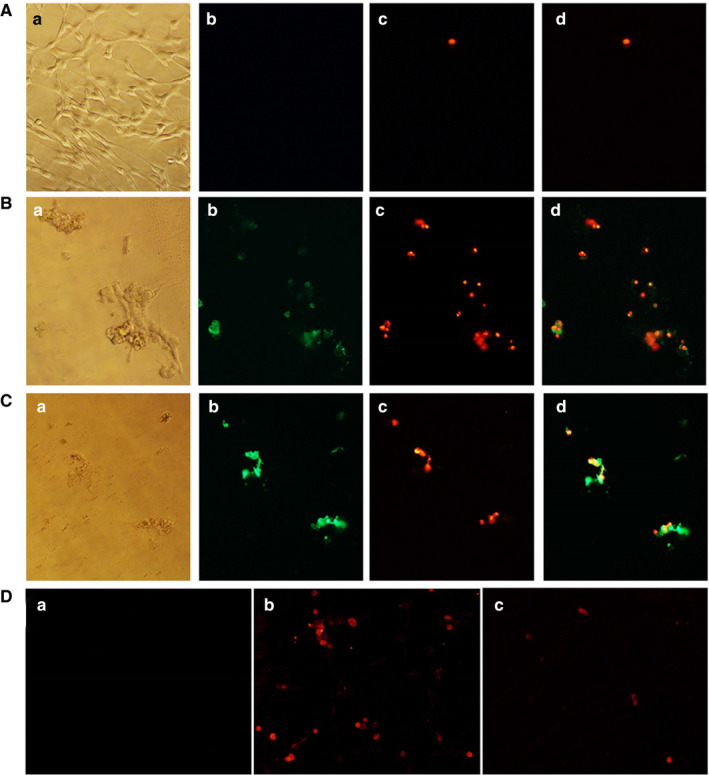
*K rotunda* and *Z mauritiana*‐mediated Ag/AgCl‐NPs induced apoptosis in GSCs. (A), (B) and (C) representing control, *K rotunda* and *Z mauritiana*‐mediated Ag/AgCl‐NPs‐treated cells respectively. Where (a) optical microscopic view and (b), (c) and (d) fluorometric view after staining with annexin V/PI and merged of these two, respectively. D, Immunofluorometric assay of *K rotunda* and *Z mauritiana*‐mediated Ag/AgCl‐NPs against GSCs. Where (a), (b) and (c) representing control and *K rotunda* and *Z mauritiana*‐mediated Ag/AgCl‐NPs‐treated GSCs. All pictures were captured at 40× magnification

After treatment of GSCs with Ag/AgCl‐NPs, cells were stained with FITC labelled annexin V and propidium iodide (PI). A significant number of early apoptotic and late apoptotic cells were observed for *K rotunda* tuberous rhizome and *Z mauritiana* fruit extract‐mediated Ag/AgCl‐NPs by fluorescence microscope (Figure 4A‐C). In the immunofluorescence assay, expression of caspase‐3 was increased due to the induction of apoptosis in the cells as presented in Figure 4D.

### 3.4 Ag/AgCl‐NPs induce modulation of gene expression

**FIGURE 5:**
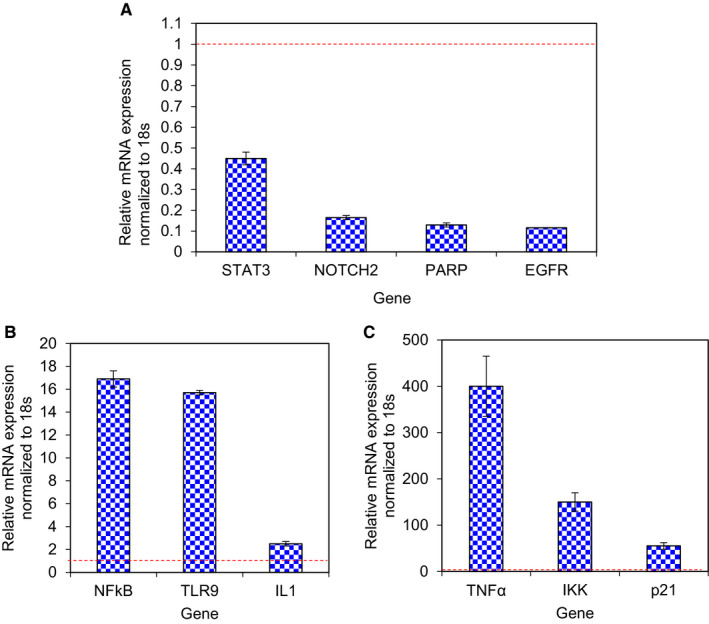
Assessment of gene expression levels. A, Expression of STAT3, Notch2, PARP and EGFR genes (B) NFκB, TLR9 and IL1 gene (C) TNFα, IKK and p21 gene after treatment of GSCs with *K rotunda‐*mediated Ag/AgCl‐NPs. Dashed line (Reddish colour) indicates 1.0 expression level

GSCs were treated with *K rotunda* tuberous rhizome‐mediated Ag/AgCl‐NPs for 48 hours to observe the expression level of genes by real‐time PCR. The levels of expressions of PARP, EGFR, NOTCH2 and STAT3 gene were decreased with the increase of NFκB, TLR9, IL1, TNFα, IKK and p21 as shown in Figure 5A‐C.

### 3.5 Cell cycle analysis

**FIGURE 6:**
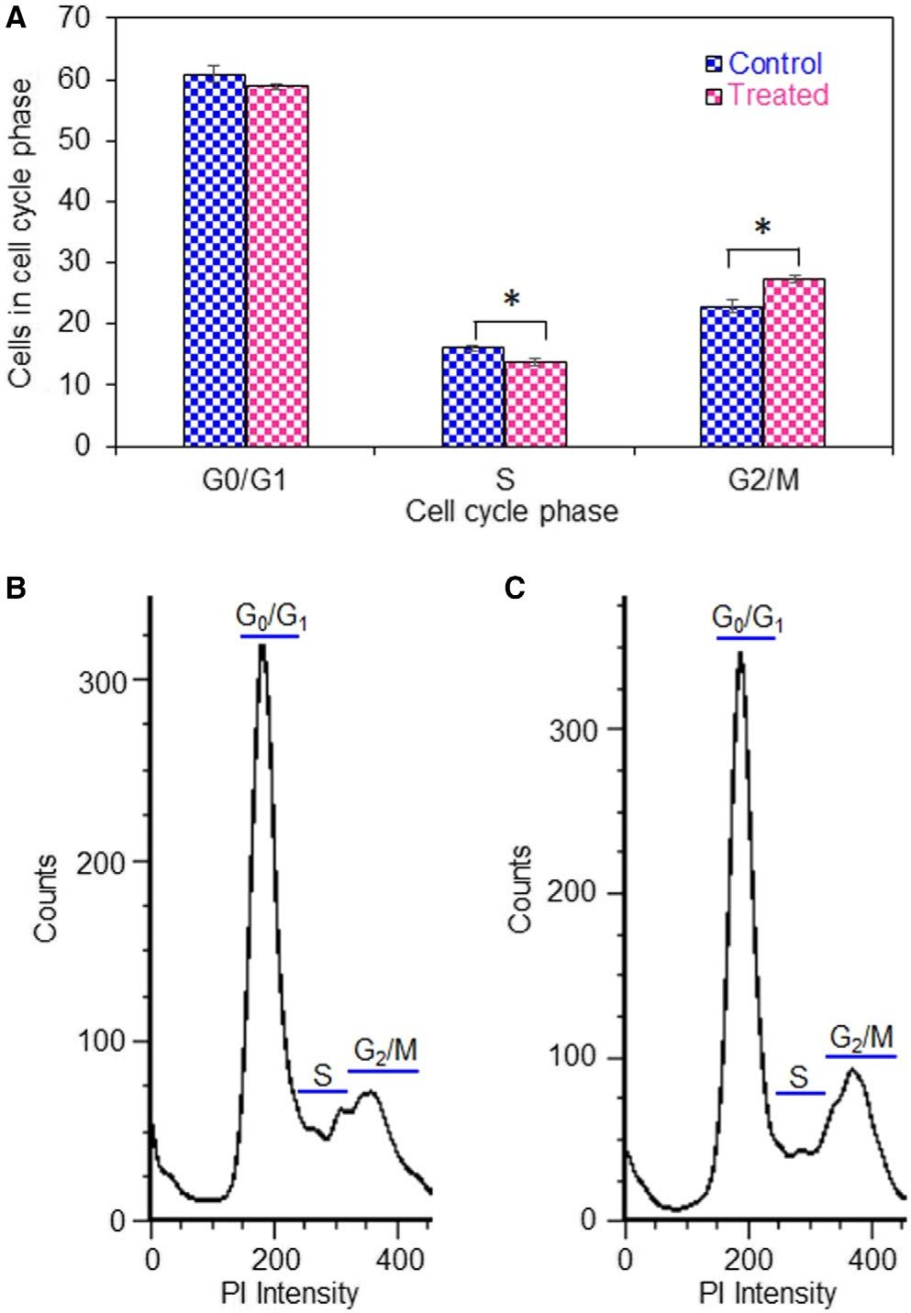
Effects of *K rotunda‐*mediated Ag/AgCl‐NPs on cell cycle distribution in GSCs. The effects of synthesized nanoparticles on the cell cycle of GSCs were evaluated by flow cytometry. A, The percentages of each cell cycle were analysed based on mean values obtained from three independent experiments. B, and C, Representing histogram of control and treated GSCs respectively. *P > .01 as compared with the control

The percentages of G_0_/G_1_, S and G_2_/M phases in control GSCs were calculated to be 60.9, 16.1 and 22.94, respectively. After treatment with *K rotunda* tuberous rhizome‐mediated Ag/AgCl‐NPs, percent of G_0_/G_1_ and S decreased to 58.9, and 13.73 respectively, consequently with the increased of G_2_/M phase to 27.37%. The above results revealed the G_2_/M phase cell cycle arrest in GSCs (Figure 6A‐C).

### 3.6 Antitumour activity against EAC cells in vivo in mice

**FIGURE 7:**
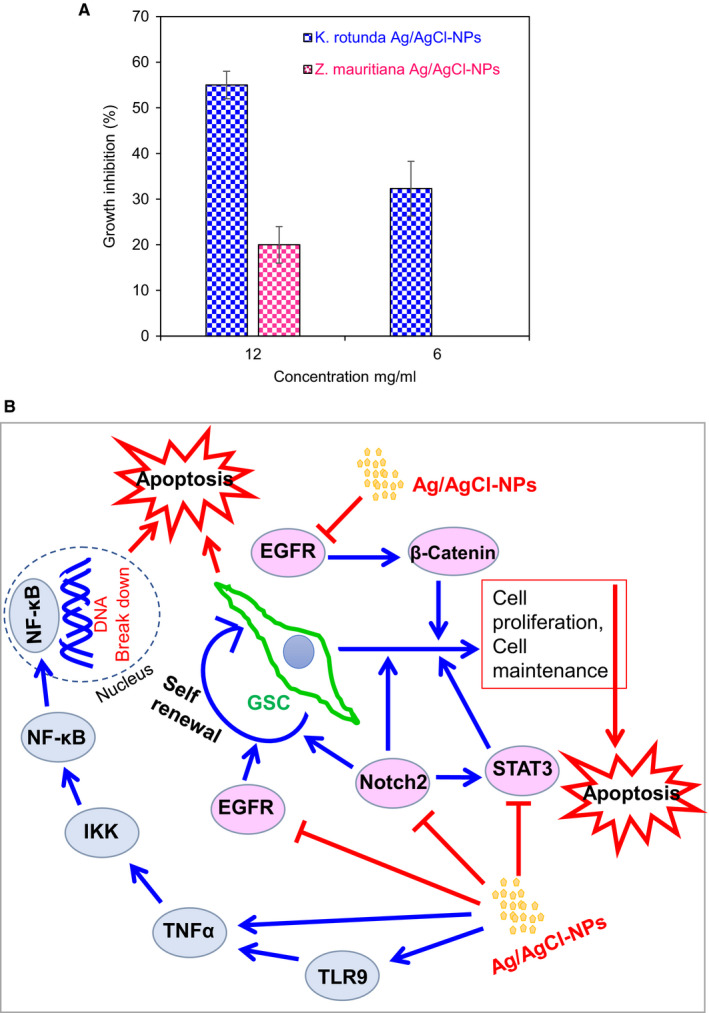
Effects of Ag/AgCl‐NPs on EAC cells and schematic representation of genes expression. A, EAC cells growth inhibition in the presence of *K rotunda* and *Z mauritiana*‐mediated Ag/AgCl‐NPs in mice. Data are expressed in mean ± SD (n = 6). B, Schematic representation of the effects of *K rotunda* tuberous rhizome‐mediated Ag/AgCl‐NPs on several genes

At the 6 mg/kg/day dose, 32.3% of growth inhibition was observed for *K rotunda* tuberous rhizome‐mediated Ag/AgCl‐NPs and no inhibition was found for *Z mauritiana* fruit extract‐mediated Ag/AgCl‐NPs. When the dose level was increased to 12 mg/kg/day, 55% and 20% of EAC cells growth were inhibited by *K rotunda* tuberous rhizome and *Z mauritiana* fruit extract‐mediated Ag/AgCl‐NPs, respectively (Figure 7A).

## 4 DISCUSSION

In the present study, Ag/AgCl‐NPs were synthesized by treating of AgNO_3_ with *K rotunda* tuberous rhizome extract. Formation of *K rotunda* tuberous rhizome extract‐mediated Ag/AgCl‐NPs was confirmed by the change of colour from transparent to deep brown and absorbance peak between 400 and 450 nm of UV‐visible spectroscopy. The highest absorbance peak at the wavelength of 437 nm was due to the surface plasmon resonance.^25^ No such type of peak was observed for *K rotunda* tuberous rhizome extract. The intensity of the absorbance was increased with the rise of AgNO_3_ concentration that could be due to the number of nanoparticle formation.

Size of the nanoparticles was observed to be spherical and highly monodispersed with an average diameter of 17 nm as estimated by TEM. Most of the plant‐mediated silver nanoparticles were reported to be spherical and various plant material act as reducing and capping agents of silver.^3^, ^4^, ^6^ The crystalline nature of Ag/AgCl‐NPs and the presence of silver and silver chloride were confirmed by XRD. The formation of AgCl‐NPs was explained by Kang et al who revealed that synthesis of AgCl‐NPs from oligomeric chitosan occurred in two steps.^26^ At first, Ag ions readily reacted with Cl ions and the amino and hydroxyl groups stabilized the AgCl‐NPs. From that study, it can be assumed that the chlorine ion of the Tris‐HCl buffer reacted with the silver ion and formed AgCl. Different materials of the *K rotunda* tuberous rhizome extract were then used to stabilize the AgCl as AgCl‐NPs. In the earlier investigation, we had also reported *Z mauritiana* fruit extract‐mediated Ag/AgCl‐NPs.^2^ From the TGA data, it was found that the nanoparticles lost their weight in three stages. The first weight loss happened due to the removal of water whereas second and third weight loss occurred for the decomposition or elimination of capping biomolecules.^27^ That designate the synthesized nanoparticles possessed high thermal stability. AFM image was used to determine the size and shape of the nanoparticles.^28^, ^29^ In the present study, it was not possible due to the agglutination of the particles on the glass surface. The topography of the agglutinated Ag/AgCl‐NPs was observed in the AFM image. Such type of agglutination is common and was observed previously.^2^, ^30^

The presence of various functional groups in *K rotunda* rhizome extract and synthesized Ag/AgCl‐NPs was identified by FTIR. The peaks obtained at 3382.73 and 3433.70 cm^−1^ in the *K rotunda* tuberous rhizome extract and Ag/AgCl‐NPs respectively might be due to the –OH stretching of alcohols and phenols or bending stretching of hydrogen‐bonded alcohols or phenols.^31^ 2929.03 and 2919.99 cm^−1^ due to the ‐CH stretching of alkanes, 1630.52 and 1637.71 for the bending vibration of the N‐H bend of 1° amines, 1384.48 and 1384.26 for N‐O group, 1061.03 and 1079.10 cm^−1^ stretching of esters.^25^, ^31^ The band observed at 620.16 and 558.11 cm^−1^ might be because of the C‐Cl stretching of alkyl chloride. From the comparative analysis, it can be suggested that *K rotunda* tuberous rhizome extract and the Ag/AgCl‐NPs share certain common functional groups. Based on the FTIR analysis, it can be assumed alkyl chloride and phenol compounds present in the extract may be involved for the formation of AgCl and capping and stabilizing agents of Ag and AgCl nanoparticles.

Cancer is one of the leading causes of death worldwide after cardiovascular diseases. There are more than 100 different types of cancer. The World Health Organization (WHO) estimated 9.6 million people deaths in 2018. Most common type of tumours of the Central Nervous System (CNS) is gliomas. Glioblastoma multiforme that is known as glioma stem cells (GSCs) as cancer stem cells (CSCs) were identified in the grade IV gliomas and the 5‐year survival of patients is below 5%. GSCs are responsible for maintaining these tumours after therapy and repopulating them after gross total resection. GSCs are regulated by various mechanisms including intrinsic and extrinsic factors. Epigenetics, metabolism and genetics are intrinsic factors while cellular microenvironment, the host immune system and niche factors are known as extrinsic factors.^32^ Recently, a number of molecular mechanisms, for example, DNA damage checkpoint, Notch‐2, NF‐kB, EZH2 and PARP have been identified that facilitated the therapeutic resistance of CSCs to cytotoxic therapies. It can be suggested that CSCs develop multiple mechanisms of resistance requiring combination of targeted agents. In the present study, *K rotunda* tuberous rhizome and *Z mauritiana* fruit extract‐mediated Ag/AgCl‐NPs were used to evaluate the anticancer mechanism against GSCs. IC_50_ values of *K rotunda* tuberous rhizome and *Z mauritiana* fruit extract‐mediated Ag/AgCl‐NPs were calculated to be 6.8 and 10.4 µg/mL, respectively. That shows *K rotunda* tuberous rhizome‐mediated Ag/AgCl‐NPs are more effective than that of the and *Z mauritiana* fruit extract‐mediated NPs against GSCs. Antiproliferative activities of the synthesized NPs were also checked against EAC cells. The IC_50_ value was calculated to be 7.85 µg/mL. On the other hand, IC_50_ value of *Z mauritiana* fruit extract‐mediated Ag/AgCl‐NPs was 84 μg/mL.^2^ The results designated that *K rotunda* tuberous rhizome‐mediated Ag/AgCl‐NPs are more effective than *Z mauritiana* fruit extract‐mediated Ag/AgCl‐NPs. *K rotunda* tuberous rhizome extract retained comparatively low antiproliferative activity. In the present, investigation induction of apoptosis in GSCs by the *K rotunda* tuberous rhizome and *Z mauritiana* fruit extract‐mediated Ag/AgCl‐NPs was checked after staining with FITC‐annexin‐V/PI. Induction of apoptosis was observed both in the early and late stages. Apoptosis was further confirmed by immunofluorescence assay.

A number of genes are related with the growth and maintenance of GSCs and their apoptotic cell death. Researchers are trying to inhibit GSCs growth by modulating expression of these genes with different compounds. It was reported that when PARP inhibitor (ABT‐888) and TMZ treated with GBM in the presence of radiation, apoptosis in GBM increased significantly.^33^ Here, expression of PARP genes was down‐regulated after treatment with *K rotunda* tuberous rhizome‐mediated Ag/AgCl‐NPs. GSCs up‐regulated a number of signalling pathways essential for maintaining neural stem cells stemness, which enables them to increase their stemness and unusual cell survival, consequently leading to tumorigenesis.^34^, ^35^, ^36^ Targeting these signalling pathways and their receptors in GSCs holds promise for glioblastoma therapy.

In mammals, four homologous proteins function as cytoplasmic receptors are known as Notch1, Notch2, Notch3 and Notch4.^37^ Notch signalling pathway is important for cell proliferation, apoptosis, stem cell maintenance and tissue homeostasis.^38^, ^39^, ^40^ In Glioblastoma tissue, astrocyte fate genes, stemness genes and anti‐apoptotic proteins are correlated with the expression level of Notch2 gene.^41^, ^42^ It was reported that lower levels of miR‐34c‐3p and miR‐34c‐5p correlate with a higher glioma grade. The over expression of miR‐34c‐3p (miRNAs) strongly inhibits glioma invasion and promotes the S‐phase arrest, increases cell apoptosis and reduces Notch2 expression.^43^ Ruan et al reported that another miRNA (miR‐181c) reduces cell invasion, cell proliferation and self‐renewal capacities of glioblastoma cell through the down‐regulation of Notch2 gene.^44^ In the present study, Notch2 gene was down‐regulated after treatment with the synthesized nanoparticles that may be a possible reason of apoptosis in GSCs.

The signal transducer and activator of transcription 3 (STAT3) is involved in the central nervous system development, immune response, stem cell maintenance and tumorigenesis. Activation of STAT3 plays important role in glioma biology.^45^ Cao et al reported that when GSCs were treated with STAT3 inhibitor (WP1066), the cell number was decreased with the induction of apoptosis.^46^ Sherry et al showed that STAT3 is needed for the proliferation and maintenance of GSCs.^47^ It had been demonstrated that Notch signalling could regulate STAT3 activation and targeting the pathway inhibits STAT3 activation, and cell growth and self‐renewal in GSCs.^46^, ^48^ In 2012, Sai et al found that a novel small molecule, WP1193, inhibitor of the JAK2/STAT3 pathway induced cell cycle arrest and apoptosis in glioblastoma stem‐like cells.^49^ Here, down‐regulation of STAT3 may also be the cause of apoptosis in GSCs. Aberrant activation of Wnt signalling in GSCs led to tumour growth through nuclear localization of stabilized β‐catenin.^50^, ^51^ EGFR activation promotes GSCs proliferation and tumorigenesis by transactivation of β‐catenin.^52^ EGFR expression was also down‐regulated in the present study which might be also another reason for GSCs growth inhibition.

Members of the NF‐κB family regulated cell invasion, cell proliferation, cell cycle progression and apoptosis as important transcription factors in tumours.^53^, ^54^, ^55^ NF‐κB (p65) pathway typically activated by phosphorylation of IκBα, which is the inhibitory subunit of NF‐κB.^56^ IκBα is phosphorylated by IKKα/β, which leads to IκBα degradation as a result p65 and p50 are released and trafficked into the nucleus where they evoke gene transcription.^56^, ^57^ TNFα is known to increase phosphorylation of IKK causing a decrease of IKBα levels and increase of nuclear p65.^58^ Toll‐like receptors (TLRs) acted for transcription of the NF‐κB gene and activated pro‐inflammatory cytokines, such as IL6 and TNFα through the adaptor molecule MyD88.^59^, ^60^ The present study clearly stated the up‐regulation of TLR9, NF‐κB, TNFα, IKK and IL1 (induced IL6) gene expression after treatment of GSCs with *K rotunda* tuberous rhizome‐mediated Ag/AgCl‐NPs. It was reported that NF‐κB retained its functions as both an anti‐apoptotic and pro‐apoptotic regulatory factor^61^ and the nature of the apoptotic stimulus regulate the pro‐apoptotic or anti‐apoptotic function.^62^ In this study, Ag/AgCl‐NPs stimulated NF‐κB to work as pro‐apoptotic gene against GSCs. The Ag/AgCl‐NPs might have induced apoptosis by the activation of cellular apoptotic pathway whereas TNFα was activated directly and/or through TLR9, which consequently activated IKK, NF‐κB and finally apoptosis happened in GSCs due to the breakdown of DNA. Cell cycle arrest at the G_2_/M phase was also observed by flow cytometry and further confirmed by several fold increase of p21 gene. Effects of the Ag/AgCl‐NPs on several genes have been diagrammatically presented in Figure 7B.

Several in vitro experiments were carried out to study the anticancer effect of AgNPs on different cancer cell lines^8^, ^10^, ^12^ but only a very few were performed in vivo in mice.^9^ To check the in vivo antitumour activity of *K rotunda* tuberous rhizome and *Z mauritiana* fruit extract‐mediated Ag/AgCl‐NPs, EAC cells were selected for its rapid proliferate in mice due to lack of H2 histocompatibility antigen.^63^ In vivo experiments stated that *Z mauritiana* fruit extract‐mediated Ag/AgCl‐NPs showed low antitumour activity against EAC cells. While, *K rotunda* tuberous rhizome‐mediated Ag/AgCl‐NPs retained strong antitumour activity. The in vivo results revealed that antitumour properties of biogenic nanoparticles vary from sample to sample for different factors those including size distribution, morphology, surface charge, surface chemistry and capping agents.^13^, ^16^

In conclusion, Ag/AgCl‐NPs were synthesized from the *K rotunda* tuberous rhizome and characterized by UV‐visible spectroscopy, TEM, XRD, TGA, AFM and FTIR *K. rotunda* tuberous rhizome and *Z mauritiana*‐mediated Ag/AgCl‐NPs inhibited GSCs growth significantly by the induction of apoptosis. Antiproliferative activity of *K rotunda* tuberous rhizome‐mediated Ag/AgCl‐NPs was due to the G_2_/M cell cycle arrest and modulation of several genes those related to GSCs cell invasion, cell proliferation, cell cycle progression and apoptosis. In vivo experiments revealed that *K rotunda*‐mediated Ag/AgCl‐NPs retained strong antitumour activity against EAC cells. For the treatment of brain tumour, further research is required to know about the adverse reaction and efficacy of the biogenic Ag/AgCl‐NPs.

## CONFLICT OF INTEREST

The authors have no conflict of interest.

## AUTHOR CONTRIBUTION

**Syed Rashel Kabir:** Conceptualization (lead); Funding acquisition (equal); Investigation (lead); Project administration (equal); Resources (equal); Software (lead); Writing‐original draft (lead). **Zhi Dai:** Investigation (supporting). **M. Nurujjaman:** Investigation (supporting). **Xiaoyue Cui:** Investigation (supporting). **A. K. M. Asaduzzaman:** Investigation (supporting). **Bin Sun:** Investigation (supporting). **Xianning Zhang:** Investigation (supporting). **Hongjuan Dai:** Investigation (supporting). **Xudong Zhao:** Funding acquisition (equal); Project administration (equal); Resources (equal).

## Supporting information

Table S1
