# Supplementary material for: Biogenic silver/silver chloride nanoparticles inhibit human glioblastoma stem cells growth in vitro and Ehrlich ascites carcinoma cell growth in vivo
Source: J Cell Mol Med. 2020 Oct 13;24(22):13223–34. doi: 10.1111/jcmm.15934 (PMC7701582; doi:10.1111/jcmm.15934)
Supplement: Supplementary file 1 — Table S1 [file JCMM-24-13223-s001.docx]

**Supplementary Table-1: List of Primers**

| **NFkB** | **F** | **CCAGTATCCCGGTCCAGCTAT** |
| --- | --- | --- |
|  | **R** | **CACGTCCAACTCACTCCAAGG** |
| **TNFα** | **F** | **ATTGCCGCAGAAAGTTCTACG** |
|  | **R** | **GTCCAGTTTCGTCTTCAGCTC** |
| 18s | F | GTAACCCGTTGAACCCCATT |
|  | R | CCATCCAATCGGTAGTAGCG |
| TLR9 | F | CTGCCTTCCTACCCTGTGAG |
|  | R | GGATGCGGTTGGAGGACAA |
| PARP | F | GGCCTCGGTGGATGGAATG |
|  | R | GCAAACTAACCCGGATAGTCTCT |
| STAT3 | F | CAGCAGCTTGACACACGGTA |
|  | R | AAACACCAAAGTGGCATGTGA |
| NOTCH2 | F | CAACCGCAATGGAGGCTATG |
|  | R | GCGAAGGCACAATCATCAATGTT |
| EGFR | F | AGGCACGAGTAACAAGCTCAC |
|  | R | ATGAGGACATAACCAGCCACC |
| P21 | F | TGCAACTACTACAGAAACTGCTG |
|  | R | CAAAGTGGTCGGTAGCCACA |
| IKK | F | CCCAACGTATGTGGGACCAAG |
|  | R | CACAGCGGTCTTTGCACTTAC |
| IL-1 | F | CATTGAGCCTCATGCTCTGTT |
|  | R | CGCTGTCTGAGCGGATGAA |
